# Polymorphic phase transition in liquid and supercritical carbon dioxide

**DOI:** 10.1038/s41598-020-68451-y

**Published:** 2020-07-17

**Authors:** Vitaliy Pipich, Dietmar Schwahn

**Affiliations:** 1 Forschungszentrum Jülich GmbH, Jülich Centre for Neutron Science (JCNS) at Heinz Maier-Leibnitz Zentrum (MLZ), Lichtenbergstraße 1, 85748 Garching, Germany; 2 Forschungszentrum Jülich GmbH, Jülich Centre for Neutron Science (JCNS-1), Wilhelm Johnen Strasse, D-52428 Jülich, Germany

**Keywords:** Physics, Chemical physics, Statistical physics, thermodynamics and nonlinear dynamics

## Abstract

We present experiments on molecular density fluctuations in liquid and supercritical (SC) CO_2_ using small-angle neutron scattering. Thermal density fluctuations in SC-CO_2_ determine susceptibility and correlation length identifying the Widom line at their maxima. Droplet formation occurs at the gas–liquid line and between 20 and 60 bar above the Widom line, the corresponding borderline identified as the Frenkel line. The droplets start to form spheres of constant radius of ≈ 45 Å and transform into rods and globules at higher pressure. Droplet formation represents a liquid–liquid (polymorphic) phase transition of the same composition but different density, whose difference defines its order parameter. Polymorphism in CO_2_ is a new observation stimulating interesting discussions on the topics of gas-like to liquid-like transition in SC fluids and polymorphism since CO_2_ represents a “simple” van der Waals liquid in contrast to water, which is the most widely studied liquid showing polymorphism in its supercooled state.

## Introduction

**Figure 1:**
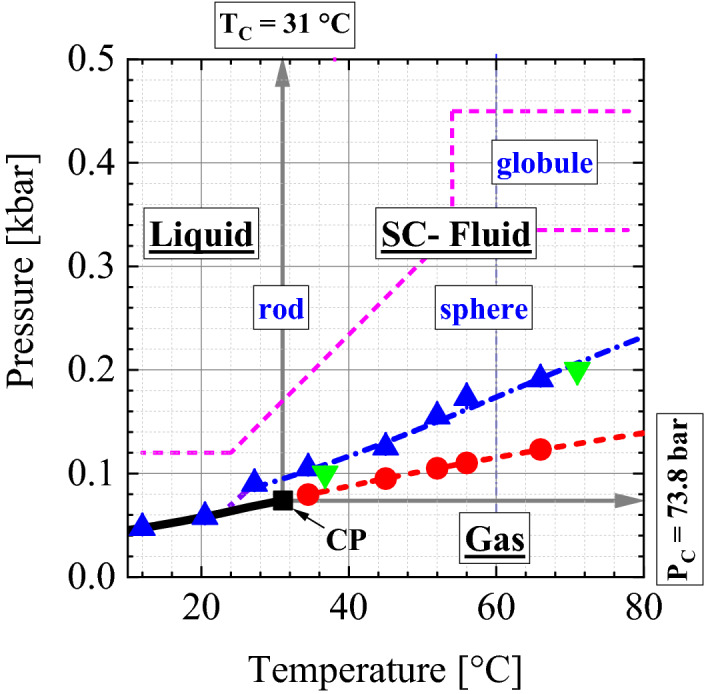
Temperature–pressure plane of the CO_2_ phase diagram. The solid line represents the gas–liquid phase boundary with the critical point (CP) at its endpoint T_C_ = 31 °C and P_C_ = 73.8 bar. The solid grey lines with an arrow represent the borderlines of gas, liquid, and supercritical (SC) fluid phases. The dashed and dashed-dotted lines, respectively, show the Widom ( ) and Frenkel [ (isotherm),  (isobar)] lines, and the symbols show the corresponding SANS values. The Widom and Frenkel lines represent the locations of the largest degree of thermal molecular density fluctuations and the onset of droplet formation, respectively. Droplets of different shapes form beyond the gas–liquid and Frenkel line representing a liquid–liquid phase transition of phases with the same composition but different molecular densities. Such phase transition obeys the class of polymorphic phase transition of one-component disordered systems such as liquids and glasses, whose most prominent representative is water.

Classical thermodynamics regards supercritical fluids (SCFs) as a homogeneous phase, which opens up a pathway to transform a gas phase into the liquid phase without passing any phase transition line^1,2^. This perception has changed in recent years as demonstrated in the pressure–temperature projection of the phase diagram of CO_2_ in Fig. 1, showing the gas–liquid phase transition line (solid line), the critical point (CP), and the Widom line in the SCF regime above CP^3–5^. The experimental points of the Widom ( ) and Frenkel [ (isotherm),  (isobar)] lines were determined from small-angle neutron scattering (SANS) in Ref.^6^ as well as in this paper. Both lines characterize different kinds of molecular density fluctuations. The Widom line (dashed line) is defined as the position of maximum strength of thermal density fluctuations expressed as susceptibility (i.e. the thermodynamic response function of the compressibility) and of the largest correlation length. Both parameters become infinite at the gas–liquid critical point^7–10^. The Frenkel line was originally defined as a dynamic borderline between gas-like and liquid-like phases on the basis of purely diffusive and diffusive plus vibrational molecular motion, respectively^11,12^. A similar borderline was proposed by Fisher and Widom claiming a “certain rough distinction between gas and liquid” on the basis of the density pair correlation function showing, respectively, a monotonic and oscillatory asymptotic decay^13^. A one-dimensional model assuming an infinite hard-core repulsion and short-range square-well attraction showed such a borderline, but this model could not be extended to higher dimensions^14^.

Quite recently, we published SANS data on SC-CO_2_ determining density fluctuations along the isothermal pathway of 45 °C from 60 to 460 bar, i.e. at T/T_C_ = 1.046 and P/P_C_ from 0.81 to 6.23^6^. We determined the Widom line at 95 bar (P/P_C_ = 1.29) and, surprisingly, found the formation of droplets of about 45 Å radius above 125 bar. i.e. 30 bar above the Widom line. These droplets only differ in number density (n) from the SCF phase, i.e. n_D_ ≠ n_F_, thereby representing a polymorphic, i.e. liquid–liquid phase transition. We identified the phase boundary of droplet formation with the Frenkel line. In the literature, such structural changes in SCFs are partly identified as the Widom^8,10^ and Frenkel lines^15,16^ (see further references in Supplementary Section B3). We report here about SANS experiments on CO_2_ in an extended temperature and pressure regime from 12 to 66 °C and from 60 to 480 bar; a result are the dots and triangles in Fig. 1 identified as Widom and Frenkel lines, respectively. Our main motivation was to explore droplet formation over a larger area above the gas–liquid as well as the Frenkel phase boundaries. We always determined droplets in their equilibrium state as the dynamics of CO_2_ is fast due to diffusion and the piston effect near the critical point (critical slowing down)^17^ (see also Supplementary Fig. B3b in Supplementary Section B). The main results of our experiments are (i) shape and size of the droplets, (ii) estimation of CO_2_ number density of droplets, as well as (iii) the characteristic of phase transition.

## Results

**Figure 2:**
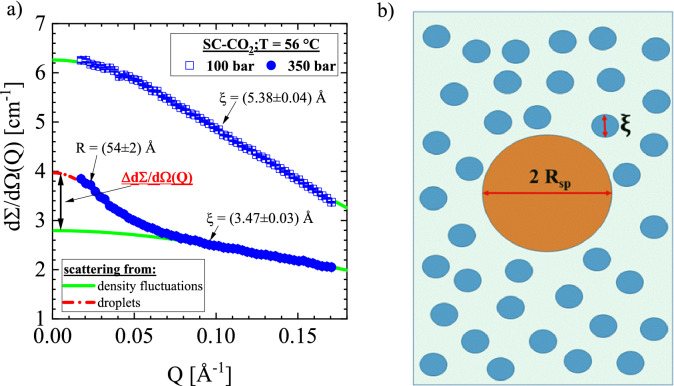
SC-CO_2_ at 56 °C: scattering cross-section and schematic image. (**a**) Scattering pattern below the Widom line (P_W_ = 110 bar) at 100 bar showing only scattering from thermal density fluctuations according to the Ornstein–Zernicke law in Eq. (2) (solid line). On the other hand, scattering at 350 bar shows scattering (dashed-dotted line) from thermal density fluctuations (solid line) and in addition from droplets (double arrow) analyzed with Eqs. (6)–(9). Both contributions superimpose incoherently, i.e. their intensities are added. The Frenkel line was determined at (173 ± 10) bar. (**b**) The schematic image shows thermal fluctuations of correlation length ξ and a droplet of radius R_sp_ in accordance with ${\text{d}}\Sigma /{\text{d}}\Omega {\text{(Q)}}$ at 350 bar in (**a**).

Before presenting the experimental results, we would like to clarify the procedure of data analysis. Figure 2 shows two scattering curves depicted as macroscopic cross-section (${\text{d}}\Sigma /{\text{d}}\Omega ({\text{Q}})$) versus the modulus of scattering vector (Q) defined in the text below in Eq. (2). Both curves were measured at two pressures of 100 bar and 350 bar (Widom line P_W_ = 110 bar) at 56 °C, which is, respectively, below and above the Frenkel line [P_Fr._ = (173 ± 10) bar]. Below the Frenkel line, we only measure thermal density fluctuations, which according to the Ornstein–Zernicke (OZ) law (Eq. 2) are described by two parameters, namely ${\text{d}}\Sigma {\text{/d}}\Omega {\text{(Q = 0)}}$ and the correlation length (ξ). ${\text{d}}\Sigma {\text{/d}}\Omega {\text{(Q = 0)}}$ is proportional to the susceptibility S(Q = 0) (Eq. 3), which itself is associated with the compressibility $\left. {\partial {\text{n}}/\partial {\text{P}}} \right|_{{\text{T,V}}}$ as outlined in Eq. (5). The scattering curve at 350 bar (solid dots) has two contributions: one from thermal density fluctuations (solid line) and the other one from droplets bar (dashed-dotted line). The droplet part of scattering was separated from the total scattering via subtraction of the thermal diffuse part and is indicated by the double arrow and analyzed with Eqs. (6)–(9).

Supplementary Section A presents all the scattering data of thermal density fluctuations together with their analysis on the basis of the corresponding scattering laws of Eqs. (2)–(5). The results are the susceptibility S(0) and correlation length ξ measured below and above CP and depicted as a function of applied pressure. Independent of SANS, S(0) was evaluated on the basis of Eq. (5) from the CO_2_ number density n(T, P) depicted as a solid line in Supplementary Fig. A2 of Supplementary Section A^18^. Both susceptibilities are in excellent agreement. In some cases, we had to slightly adjust the temperature of the SANS measurements by a few degrees in order to obtain the same position of the maxima in accordance with the Widom line.

Supplementary Section B shows the SANS data of droplet formation above the gas–liquid line (Supplementary Fig. B1 in Supplementary Section B) and Frenkel line (Supplementary Fig. B3 in Supplementary Section B). All the figures present ${\Delta d}\Sigma {\text{/d}}\Omega {\text{(Q)}}$ (see Fig. 2) together with the fitted curves depicted as solid lines. The droplets are always in their equilibrium state as was tested for several temperatures as shown in Supplementary Fig. B3b of Supplementary Section B and the measurements were performed along the isothermal pathway with ascending and descending pressure. Fit parameters are ${\Delta d}\Sigma {\text{/d}}\Omega {(0)}$ as well as the radius of spheres (R_sp_) and cross-section (R_rod_) and length (L_rod_) of the rods. The scattering patterns were fitted with Eq. (6) together with the corresponding form factor for spheres (Eq. 7), rods (Eq. 8), and in the case of globules with Eq. (9). Supplementary Section C presents all these parameters in Supplementary Tables C1–3.

**Figure 3:**
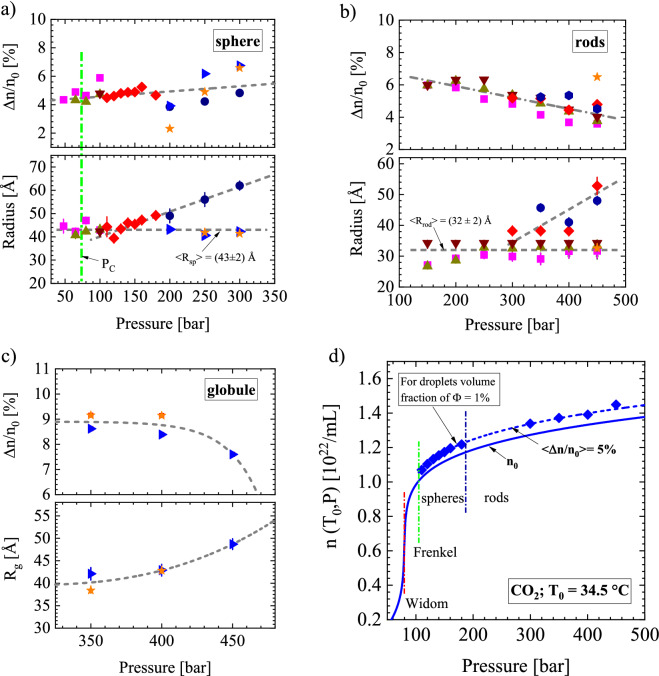
Relevant parameters of droplets. The parameter $\Delta {\text{n/n}}_{0}$ represents the relative change of CO_2_ number density of the droplets with respect to the fluid number density n_0_ assuming a droplet volume fraction of Φ = 1%. $\Delta {\text{n/n}}_{0}$ is plotted as a function of pressure. The symbols correspond to the following temperatures: 12 °C ( ), 20.5 °C ( ), 27.2 °C ( ), 34.5 °C ( ), 52 °C ( ), 56 °C ( ), 66 °C ( ). (**a**) In the lower part of the figure, the radius of all the observed spherical droplets analyzed with the form factor of spheres in Eq. (7). Whereas $\Delta {\text{n/n}}_{0}$ increases slightly with an overall average value of (1.67 ± 0.06) %, the radius splits into two parts above 130 bar. The radii below 56 °C increase linearly later transforming into rod-like droplets, whereas the radii at 56 °C and 66 °C remain constant forming a globular morphology at higher pressure. (**b**) $\Delta {\text{n/n}}_{0}$ and radius of the rod cross-section. $\Delta {\text{n/n}}_{0}$ shows a slightly linear decline with pressure, which might indicate reduced droplet volume fraction. An appreciably larger cross-section of the rods (R_rod_) is determined at higher pressure for 34.5 °C and 52 °C. (**c**) $\Delta {\text{n/n}}_{0}$ and the radius of gyration (R_g_) of the globular droplets declines and increases slightly, respectively. (**d**) Number density at T = 34.5 °C versus pressure (taken from Ref.^18^). For droplets assuming Φ = 1% volume fraction, an averaged 5% enhanced number density is obtained. In the case of Φ = 10%, a $\sqrt {{0}{\text{.1}}}$ smaller value of $\Delta {\text{n/n}}_{0}$ of ~ 1.6% is obtained.

SANS provides droplets of three different shapes as depicted in the phase diagram of Fig. 1. In the first stage, droplets always form a spherical shape before they transform into rods or globules at higher pressure. The lower part of Fig. 3a shows the radii of the spherical droplets versus pressure. An average < R_sp_ > = (43 ± 2) Å is determined for all temperatures below ~ 130 bar and up to 300 bar for 56 and 66 °C. At 34.5 and 52 °C, R_sp_ linearly increases up to ~ 60 Å above 130 bar. It may be interesting to note that spherical droplets which increase linearly and which remain constant in size later form rods and globules, respectively (Fig. 3a).

The lower part of Fig. 3b depicts the radii of the rod cross-section (Fig. 1). An average < R_rod_ > = (32 ± 2) Å is determined for all temperatures except for 34.5 °C and 52 °C, showing enlarged radii up to ~ 50 Å above 300 bar. The length of the rods (L_rod_) could only be determined with large error bars and sometimes even not then if L_rod_ became too large to fulfill the condition of L_rod_ < 1/Q for the smallest Q (Supplementary Figs. B1 and B2 and Supplementary Table C2 in Supplementary Sections B and C). However, this deficiency does not affect the determination of R_rod_ as both parameters (R_rod_ and L_rod_) are decoupled as seen from the corresponding form factor in Eq. (8) [Ref.^19^ (p 33)]. The lower part of Fig. 3c depicts the radius of gyration R_g_ of the globules formed at the higher temperatures of 56 and 66 °C and pressure of 350 bar. R_g_ increases with pressure from about 40 to 49 Å when the pressure increases from 350 to 450 bar. Assuming spherical globules, the R_g_ corresponds to a radius R_sp_ between 52 and 63 Å according to ${\text{R}}_{{{\text{sp}}}} \,{ = }\,\sqrt {5/3} \,{\text{R}}_{{\text{g}}}$[Ref.^21^ (p 159)].

Figure 3a–c also presents an estimate of the difference of the CO_2_ number densities of droplet and fluid (Δn) normalized to the number density of the fluid (n_0_), i.e. $\Delta{\text{n/n}}_{0}$ as determined with Eq. (1). $\Delta{\text{n/n}}_{0}$ is determined from the ratio of the second moment Q2 [see Eq. (10) and explanatory

1 $$ \Delta {\text{n/n}}_{{0}} \, = \,\sqrt {{\text{Q2/}}\left( {{\text{2}\pi}^{{2}} \,  \times \,\left( {1 - \Phi } \right)\Phi \times \rho_{0}^{{2}} {\text{(P,T)}}} \right)} $$

text] divided by $2\pi^{2} \, \times \,\left( {1 - \Phi } \right)\,\Phi \, \times \,\rho_{{0}}^{{2}} {\text{(P,T)}}$. The parameter ρ_0_ represents the coherent scattering length density of the fluid evaluated according to ${\text{b}}_{{{\text{CO}}_{{2}} }} {/}\Omega$ (Table 1). The problem is that the droplet volume fraction Φ is unknown. The smallest $\Delta {\text{n/n}}_{0}$ would be derived for a volume fraction of Φ = 0.5 delivering a maximum value of Φ(1 − Φ) = 0.25. However, we can reliably assume a sufficiently small droplet volume fraction, as the scattering patterns in Supplementary Figs. B1 and B2 of Supplementary Section B were reliably fitted with the corresponding form factors (F(Q)) of spheres and rods in Eqs. (6–8), which means negligible interference effects of scattering from different droplets. Refs.^20^ (p 71) and ^21^ (p 172) demonstrate the degree of interference on scattering intensity for hard spheres. A droplet volume fraction of 2% shows a ~ 10% smaller intensity at Q ~ 0 in comparison to the expected value of the form factor. We therefore assume a droplet concentration of Φ ≪ 1, i.e. $\,\left( {1 - \Phi } \right)\,\, \cong \,1$ and $\cong \,\Phi \,(\Delta {\text{n/n}}_{{0}} {)}^{{2}} .$

**Table 1:** Parameters of CO_2_ relevant for SANS such as molecular volume (Ω), coherent scattering length (${\text{b}}_{{{\text{CO}}_{{2}} }}$) and incoherent cross-section (dΣ/dΩ_inc_) with a maximum at 500 bar.

Molecule	Molar mass (g/mol)	Ω (10^–23^ cm^3^)	${\text{b}}_{{{\text{CO}}_{{2}} }}$ (10^–12^ cm)	k_B_ (10^–23^ Nm/K)	dΣ/dΩ_inc_ (cm^−1^)
CO_2_	44.01	1.77	1.826	1.3807	≅ 1 × 10^–6^ at 500 bar

In order to obtain a reasonable idea of the molecular number densities for droplets and fluid, we evaluated $\Delta {\text{n/n}}_{0}$ on the basis of Eq. (1) assuming a constant droplet volume fraction. For Φ = 1%, we obtain a $\Delta {\text{n/n}}_{0}$ in the order of 5% for the spherical and rod-like droplets, whereas between 9 and 8% is obtained for the globules as shown in the upper part of Fig. 3a–c and compiled in Supplementary Tables C1–C3 of Supplementary Section C. A ten times larger Φ of 10% droplet volume fraction would lead to a ${1/}\sqrt {{10}}$ smaller $\Delta {\text{n/n}}_{0}$ of ~ 1.6% and 2.7% for spheres/rods and globules, respectively. It seems remarkable that $\Delta {\text{n/n}}_{0}$ shows rather constant values, which might in part be based on the assumption of constant droplet volume fraction. Figure 3d shows as an example the CO_2_ number densities of droplets and fluid at T = 34.5 °C as a function of pressure. The droplets display an averaged enhanced n(T_0_, P) of about 5%. The assumed low droplet volume fraction of 1% has no measurable influence on the number density n_0_ of the fluid.

**Figure 4:**
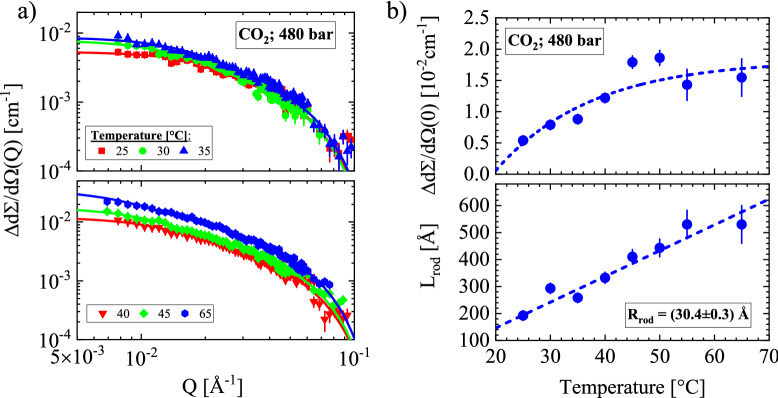
(**a**) Scattering patterns show rod-like droplets for all temperatures. (**b**) A linearly increasing length and constant cross-section of radius of (30.4 ± 0.3) Å is found with increasing temperature. This experiment was extended to 20 m sample-to-detector distance thereby allowed the determination of larger L_rod_.

We now present SANS data along the isobaric pathway of the maximal applied pressure of 480 bar (Fig. 4). Measurements along 100 and 200 bar give results consistent with the corresponding isothermal measurements as shown from the plotted triangles identifying the Frenkel line and shape of the droplets (Fig. 1). The 480 bar isobaric pathway covers an area of phase diagram not covered by earlier experiments. The form factor of rods in Eq. (8) successfully fitted all scattering patterns in the upper and lower part of Fig. 4a, as seen from the coincidence of the fitted lines and corresponding scattering data. Figure 4b depicts the parameters of the fits delivering a constant cross-section of R_rod_ = (30.4 ± 0.3) Å and a linearly increasing length of the rods and scattering intensity with temperature at Q = 0.

## Conclusion

The observation of droplets of different shapes in the liquid and supercritical state of CO_2_ is the essential result of the present work. The droplet formation represents a liquid–liquid or polymorphic transition of phases of the same composition but with slightly different number density. Such phase transition in disordered one-component liquids and glasses is uncommon. Molecular density is the order parameter and driving force of polymorphic phase transition in contrast to the chemical potential in multicomponent systems as reviewed for several liquids and glasses^22–25^. Polymorphic transition has been extensively explored in supercooled liquids, whose most prominent example is water since Stanley’s group showed evidence of two distinct liquid phases and a second critical point on the basis of molecular dynamic (MD) simulation^26,27^. To our knowledge, the underlying basic mechanism of polymorphic phase transition is not yet understood. In Supplementary Section B4 we give further examples from literature such as about the polymorphism of phosphorous, which is accompanied by a reversible structural change of the phosphorous molecules^28^; such a structural change of CO_2_ is not known to us in the studied pressure and temperature range.

Our SANS data on polymorphism in liquid and supercritical CO_2_ yield less complex results. The phase boundaries of the liquid–gas and Frenkel lines show no distinction with respect to polymorphism. In a first step, spherical droplets of dilute concentration, constant radius and increasing ${\Delta d}\Sigma {\text{/d}}\Omega {(0)}$ with pressure are formed (Fig. 3a and Supplementary Figs. B1–B3 in Supplementary Section B), which in a second step transform into droplets of rod-like shape and globular morphology at increasing pressure. Spherical droplets show no coarsening below ~ 130 bar (Fig. 3a) and the cross-section of the rod-like droplets below ~ 300 bar (Fig. 3b) only shows increasing length L_rod_ with pressure (Fig. 4b and Supplementary Figs. B1, B2 in Supplementary Section B). There is no indication of droplet nucleation and coarsening nor of spinodal decomposition indicating an unstable regime and a second critical point^29^. Small surface tension in supercritical fluids particularly near the critical point might explain the absent of droplet coarsening^1^. On the other hand, we expected formation of rods instead of spheres at the lower pressure fields just above the gas–liquid, critical point, and Frenkel line. We cannot exclude percolation in the high-pressure regime of rods, when L_rod_ becomes too large to be determined with SANS (Supplementary Fig. B2d, Supplementary Table C2 in Supplementary Sections B and C).

**Figure 5:**
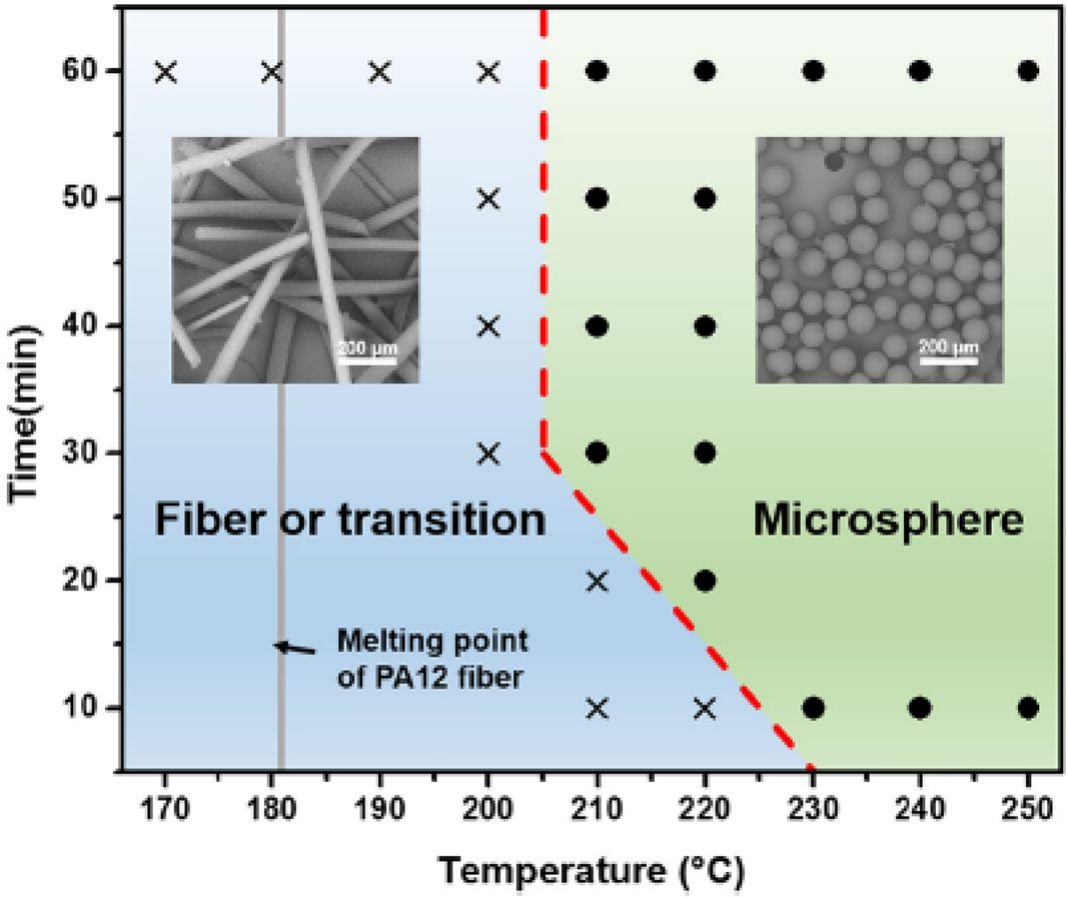
Temperature–time morphology diagram illustrating the sample morphology of PA12 (Polyamide) fibers (D_0_ = (32.6 ± 3.6 μm) after thermal treatment in the PEO (Poly(ethylene oxide) matrix. The cross represent fiber or beats-on-string morphologies and the circles represent microsphere morphologies. The grey line shows the melting point of PA12 fiber. [Figure and text from Ref.^33^ (Fig. 6)].

The phase diagram in Fig. 1 shows the transformation of rods to spheres with declining pressure or increasing temperature. The Plateau-Rayleigh instability criterion for systems displaying a large ratio of surface to volume might explain this transformation process [Ref.^30^ (Section IV. A)]. Rods of large length become unstable for fluctuations along the cylinder axis with wavelengths larger than the circumference of the rod, i.e. λ_rod_ > 2π R_rod_. Sinusoidal undulations of the surface reduce the free energy and eventually result in a series of droplets. The Rayleigh mode, λ_R_ = 9.01 R_rod_ (x_R_ = (2π/λ_R_) R_rod_ = 0.697), is the fastest growing mode and dominates the undulation^31^. The breakup of the rods occurs with the “third of the principle frequency of the Rayleigh mode” and delivers λ_R_ = 6 R_sp_ and therefore R_sp_/R_rod_ = 1.5 [Ref.^32^ (p 427)]. The Plateau-Rayleigh instability has many technical applications. An example in Fig. 5 has some similarity to our experiments showing the transformation of polyamide fibers to near-spherical particles at high temperature. Our experiments indicate a ratio of the radii of spherical and rod cross-section droplets in the range of < R_sp_ > / < R_rod_ > = (1.34 ± 0.11) (Fig. 3a, b, Supplementary Table C2 in Supplementary Section C), which we interpret as the stability condition for rods.

From the outline above, it might be not surprising to read in a recent article^34^ that on the basis of thermodynamic and structural properties a “unique and sharp separatrix” which distinguishes liquid-like and gas-like phases is “not supported” in supercritical water and other SC fluids. “Percolation may be only relevant in water due to H-bond network clustering”. These statements are supported by determinations of the Frenkel line of SC-CO_2_ on the basis of MD simulations in Refs.^12,35,36^ providing different results and thus being inconsistent with the findings presented in Ref.^6^ (Fig. 1). The same is true of the percolation line predicted by Skvor et al. correlating “simple SC Lennard–Jones (LJ) and square-well (SW) fluids with the maximum of isothermal compressibility”^37^, which, however, is attributed to the Widom line as shown in Supplementary Section A. The conclusion of Ref.^34^ is consistent with the results of the present article, namely that the Frenkel line represents the borderline of the polymorphic phase transition. This observation may provide a new basis for a discussion of the Frenkel line in SCFs.

## Methods

### Experimental equipment

The SANS experiments were performed at KWS 1 operated by JCNS at MLZ (FRM II) in Garching at a sample-to-detector distance of 1.70 m and a few experiments were performed at 7.70 and 20 m in order to achieve smaller scattering angles^38^. The neutron wavelength was 5 Å with a distribution of Δλ/λ = 10%. We designed the pressure cell especially for the SANS experiments. Two sapphire windows of 4 cm in diameter were used for the neutrons to pass through and 0.4 cm thickness for the gas. This cell allows pressures of up to 500 bar. Temperature and pressure show an estimated absolute error of ± 1 K and ± 2 bar. The SANS data were corrected for background scattering and detector efficiency and were calibrated in absolute units using a secondary standard.

Carbon dioxide (type 5.3) of purity better than 99.9993% was purchased from Linde AG (Munich, Germany). Relevant parameters of CO_2_ for the neutron scattering experiments are compiled in Table 1. The co-volume of CO_2_ molecules is related to the van der Waals parameter b and is approximately four times larger than the molecular volume Ω [Ref.^39^ (Chapter 10.3)]. The coherent scattering length was determined from the corresponding values of carbon and oxygen given in Ref.^40^ according to ${\text{b}}_{{{\text{CO}}_{{2}} }} \,{ = }\,{\text{b}}_{{\text{C}}} \,{ + }\,{\text{2b}}_{{\text{O}}}$. The incoherent scattering, dΣ/dΩ_inc_, is negligible (Table 1). We furthermore subtracted inelastic scattering from the experimental dΣ/dΩ(Q) as necessary for liquids, which will be discussed in the next section. In our earlier study of SC-CO_2_ at 45 °C, we neglected this correction thereby indicating a larger n(P) beyond the Frenkel line^6^.

### Theoretical background of scattering laws

Thermal density fluctuations in fluids such as SC-CO_2_ give rise to neutron scattering as described by the Ornstein–Zernike (OZ) law in Eq. (2).

2 $$ {\text{d}}\Sigma {\text{/d}}\Omega {\text{(Q)}}\, = \,{\text{d}}\Sigma {\text{/d}}\Omega {(0)}/\left[ {1\, + \,\left( {\xi \,Q} \right)^{2} } \right] $$

The differential macroscopic cross-section, ${\text{d}}\Sigma {\text{/d}}\Omega {\text{(Q)}}$, represents the scattered intensity per unit volume in units of cm^−1^. In the case of isotropic scattering as in the present experiment, ${\text{d}}\Sigma {\text{/d}}\Omega {\text{(Q)}}$ is a function of the modulus of the scattering vector $Q$ determined as $\left| Q \right|\,{ = }\,\left( {4\pi /\lambda } \right)\,\sin \left( {\delta /2} \right)$ from the neutron wavelength (λ) and the scattering angle (δ). The OZ law is determined by two parameters, namely, ${\text{d}}\Sigma {\text{/d}}\Omega {\text{(Q)}}$ and the correlation length ξ of the thermal fluctuations. Fluids show quasielastic scattering and no elastic scattering is observed as in crystals with their fixed atomic positions. The CO_2_ fluid obeys the class of simple liquids dominated by coherent scattering (see Table 1). Coherent scattering is expressed by the structure factor S(Q), which gives information about the relative positions of the molecules in the liquid. S(Q) is determined by integrating the scattering function S(Q,ω) over the change neutron energy $\hbar \omega$, i.e. ${\text{S}} (Q)\, = \,\int_{ - \infty }^{\infty } {S(Q,\omega )d\left( {\hbar \omega } \right)}$. Experimentally, however, the macroscopic cross-section ${\text{d}}\Sigma {\text{/d}}\Omega {\text{(Q)}}$ is determined in units of cm^−1^ representing integration over the neutron energies scattered at constant scattering angles thereby representing the static approximation [Ref.^41^ (Sect. 4.8)]. The relationship of ${\text{d}}\Sigma {\text{/d}}\Omega {\text{(Q)}}$ and S(Q) in Eq. (3) contains the correction term f_p_(Q) introduced by Placzek as discussed in Refs.^41^ (Eq. 5.39) and ^42^ (Eq. 6) and expressed in Eq. (4). The parameters are neutron (m) and molecular

3 $$ {\text{d}}\Sigma {\text{/d}}\Omega {\text{(Q)}}\, = \,{\text{K}}  \times \,\left[ {{\text{S(Q)}}\,{ + }\,f_{P} (Q)} \right] $$

4 $$ {\text{f}}_{{\text{P}}} {\text{(Q)}}\,{ = }\,\frac{{\text{m}}}{{{\text{2M}}}}\,\left[ {\frac{{{\text{k}}_{{\text{B}}} {\text{T}}}}{{\text{E}}}\, - \,\left( {{1}\,{ + }\,{0}{\text{.5}}\,\frac{{{\text{k}}_{{\text{B}}} {\text{T}}}}{{\text{E}}}} \right)\,\frac{{{\text{Q}}^{{2}} }}{{{\text{k}}^{{2}} }}} \right] $$

mass (M), yielding a ratio of m/(2 M) = 0.0115, neutron energy (E) for 5 Å yielding a ratio of k_B_T/E = 9.03 for 70 °C, and (Q/k)^2^ = 6.3 × 10^–3^ determined from the module Q of the scattering vector and neutron wavelength λ = 5 Å (k = 2π/λ = 1.26 Å^-1^). For T = 70 °C, f_P_(Q) = 0.104 is evaluated with a negligible maximal correction of 4 × 10^–4^, which becomes 0.086 in the case of 10 °C, which is about the temperature interval of our experiments. The contrast factor ${\text{K}}\,= \,{\text{n(P)}}\, \times  \,\left[ {{\text{b}}_{{{\text{CO}}_{{2}} }} } \right]^{{2}}$ describing the interaction of neutrons and atoms of CO_2_ is determined from the number density n(P) and coherent scattering length of the CO_2_ molecule (Table 1 and Ref.^40^).

The present experiment was mainly performed at constant volume (V) and temperature (T). This means that S(Q) results from fluctuations of the number density n(T, P) of CO_2_ molecules as shown in Eq. (5) for the relationship between S(Q = 0) and $\,\partial {\text{n/}}\partial {\text{P}}\left| {_{{\text{T,V}}} } \right.\,$ representing the fluctuation dissipation theorem as outlined in Ref.^39^ (p’s 103 and 337) and in Ref.^41^ (p 89). S(Q = 0) represents

5 $$ {\text{S(0)}}\,{ = }\,\left\langle {{\Delta N}^{{2}} } \right\rangle {\text{/ < N > }}\,{ = }\,{\text{k}}_{{\text{B}}} {\text{T}}\,\frac{\partial n}{{\partial P}}\left| {_{{\text{T,V}}} } \right.\, $$

a susceptibility which for molecular gases and fluids is determined by the mean square deviation of molecular number N as well as the product of the Boltzmann constant (k_B_), absolute temperature (T) and the first derivative of the number density n(P) (= < N > /V) with respect to pressure. This means that S(0) can be evaluated on the basis of the number density n(T, P) at constant T known from the equation of state determination such as found in Ref.^18^.

Beyond the Frenkel line, we observe the formation of larger scattering units which were identified as droplets^6^ and analyzed according to Eq. (6) as the product of the scattering at Q = 0, ${\text{d}}\Sigma {\text{/d}}\Omega {(0)}$, and the form factor F(Q) of spheres of radius R_sp_ and randomly oriented rods of

6 $$ \frac{{{\text{d}}\Sigma }}{{{\text{d}}\Omega }}{\text{(Q)}}\,{ = }\,\frac{{{\text{d}}\Sigma }}{{{\text{d}}\Omega }}{(0)}\, \times \,F{\text{(Q)}} $$

length L_rod_ and radius of cross-section R_rod_ as expressed in Eqs. (7) and (8), respectively^19^.

7 $$ F_{{{\text{sp}}}} {\text{(Q)}}\,{ = }\left[ {{3}\frac{{{\text{sin(QR}}_{{{\text{sp}}}} {)} - {\text{(QR}}_{{{\text{sp}}}} {\text{)cos(QR}}_{{{\text{sp}}}} {)}}}{{{\text{(QR}}_{{{\text{sp}}}} {)}^{{3}} }}} \right]^{{2}} $$

8 $$ F_{{{\text{rod}}}} {\text{(Q)}}\,{ = }\frac{{1}}{{2}}\,\int_{ - 1}^{{1}} {{\text{dz}}} \,\left[ {\frac{{{\sin}\left[ {\left( {{\text{QL}}_{rod} {/2}} \right){\text{z}}} \right]}}{{\left( {{\text{QL}}_{rod} {/2}} \right){\text{z}}}}} \right]^{{2}} \,\left[ {\frac{{{\text{2J}}_{{1}} \left( {{\text{QR}}_{{{\text{rod}}}} \sqrt {{1} - {\text{z}}^{{2}} } } \right)}}{{{\text{QR}}_{{{\text{rod}}}} \sqrt {{1} - {\text{z}}^{{2}} } }}} \right]^{{2}} $$

Another scattering law is formulated in Eq. (9) as a combination of Guinier’s and Porod’s laws

9 $$ \frac{{{\text{d}}\Sigma }}{{{\text{d}}\Omega }}{\text{(Q)}}\,{ = }\,\frac{{{\text{d}}\Sigma }}{{{\text{d}}\Omega }}{\text{(0) exp(}} - {\text{u}}^{{2}} {\text{/3) + P}}_{4} \left[ {\left( {{\text{erf}}\left( {{\text{u/}}\sqrt {6} } \right)} \right)^{{3}} {\text{/Q}}} \right]^{4} $$

representing a convenient form to analyze unspecific scattering patterns over a large Q regime^43^. The parameter R_sp_ represents the radius of the spherical droplet, u = R_g_Q the product of Q and the radius of gyration R_g_, ${\text{d}}\Sigma {\text{/d}}\Omega {(0)}\,{ = }\,\Phi \,{\text{V}}_{{\text{D}}} \,\Delta \rho^{{2}}$, and ${\text{P}}_{4} \,{ = }\,2\pi \,S_{{\text{D}}} \,\Delta \rho^{{2}}$ the scattering at Q = 0 and the Porod constant in the case of a smooth particle surface, respectively^19^. Both parameters determine the droplet volume fraction (Φ), volume (V_D_) and total surface (S_D_) per unit volume. The scattering contrast ($\Delta \rho^{2}$) is determined from the difference in the coherent scattering length densities of the droplet (D) and SCF (F), i.e. $\Delta \rho \,{ = }\,\left[ {{\rho }_{{\text{D}}} \, - {\rho }_{{\text{F}}} } \right]$^40^. For CO_2_, $\rho_{{\text{D,F}}} \, = \,\,{\text{n}}_{{\text{D,F}}} \, \times \,{\text{b}}_{{{\text{co}}_{{2}} }}$ [n_D,F_ is the number density of CO_2_ in droplet (D) and fluid (F) phase] and therefore the simplified expression $\Delta \rho \, = \,\rho_{{\text{F}}} \,\left[ {\Delta {\text{n/n}}_{{\text{F}}} } \right]\,{ = }\,{\text{b}}_{{{\text{CO}}_{{2}} }} \,\Delta {\text{n}}$ is obtained.

Information about the droplet volume fraction (Φ) and the difference in the molecular number density of droplet (n_D_) and fluid (n_F_), i.e. Δn, is obtained from the second moment of ΔdΣ/dΩ(Q), i.e. ${\text{Q2 } = \text{ }}\int {{\text{Q}}^{{2}} \Delta {\text{d}}\Sigma {\text{/d}}\Omega {\text{(Q)}}} \,{\text{dQ}}$, which according to Eq. (10) is related to the product of the droplet

10 $$ {\text{Q2 = 2}\pi }^{{2}} \,\Phi (1 - \Phi )\Delta \rho^{{2}} $$

volume fraction (Φ) and Δn^2^, i.e. ${\text{Q2}}\,{/}\,\left( {{\text{2}\pi}^{{2}} \,{\text{b}}_{{{\text{CO}}_{{2}} }}^{{2}} } \right)\, =  \,\Phi (1 - \Phi )\,\Delta {\text{n}}^{{2}} \, \cong \,\,\Phi \,\Delta {\text{n}}^{{2}}$. The same result is derived from ${\text{d}}\Sigma {\text{/d}}\Omega {(0)}\,{/}\left( {{\text{b}}_{{{\text{CO}}_{{2}} }}^{{2}} \times {\text{V}}_{{\text{D}}} } \right)\,{ = }\,\,\Phi \,\Delta {\text{n}}^{{2}}$ or ${\text{d}}\Sigma {\text{/d}}\Omega {(0)}\,{/}\left( {{\text{b}}_{{{\text{CO}}_{{2}} }}^{{2}} \times {\text{V}}_{D}^{2} } \right)\,{ = }\,\,n_{D} \,\Delta {\text{n}}^{{2}}$ yielding the droplet number density (n_D_). The linear dependence of n_D_ or Φ arises from the incoherent superposition of scattering from the droplets of dilute concentration and $\Delta {\text{n}}^{{2}}$ from the scattering contrast.

## Supplementary information

Supplementary information.
