# Supplementary material for: Polymorphic phase transition in liquid and supercritical carbon dioxide
Source: Sci Rep. 2020 Jul 17;10:11861. doi: 10.1038/s41598-020-68451-y (PMC7367860; doi:10.1038/s41598-020-68451-y)
Supplement: Supplementary file 1 — Supplementary information. [file 41598_2020_68451_MOESM1_ESM.docx]

Supplementary information: Polymorphic phase transition in liquid and supercritical carbon dioxide

Vitaliy Pipich^1^ and Dietmar Schwahn^2,*^

^1)^ Forschungszentrum Jülich GmbH, Jülich Centre for Neutron Science (JCNS) at Heinz Maier- Leibnitz Zentrum (MLZ), Lichtenbergstraße 1, D-85748 Garching

^2)^ Forschungszentrum Jülich GmbH, Jülich Centre for Neutron Science (JCNS-1)**,** Wilhelm-Johnen-Straße, D-52428 Jülich / Germany

^*)^ Corresponding author:

Dietmar Schwahn (d.schwahn@fz-juelich.de)

**A. SANS data on thermal fluctuations of the CO_2_ number density**

*A1. Thermal fluctuations - Gas-liquid transition*

The SANS data in Fig. A1 cover the gas and liquid regime below T_C_ of the CO_2_ phase diagram (Fig. 1). The plot of $\text{dΣ/dΩ}^{\text{-1}}\text{(Q)}$ versus Q^2^ in Fig. A1a represents a Zimm representation,

| a) | b) |
| --- | --- |
| c) | d) |
| **Fig. A1.** Thermal fluctuations of number density in the gas-liquid regime. a) SANS scattering data in a Zimm representation at 12 °C and between 30 and 450 bar. b) - d) Susceptibility (S(0)) and correlation length (ξ) of thermal fluctuations at 12, 20.5, and 27.2 °C versus pressure. The maximum of S(0) corresponds to the gas-liquid transition line. The green dashed-dotted line corresponds to the borderline of droplet formation. | |

describing the scattering of thermal density fluctuations as a straight line in accordance with Eq. (1). Fig. A1a shows scattering data measured along the isothermal pathway at 12 °C of hydrostatic pressure fields between 30 and 450 bar. Below the gas-liquid transition line at P_G-L_ = 47.3 bar, the data follow straight lines over the whole Q range, whereas deviations from a straight line at low Q are observed above P_G-L_ due to the formation of larger objects as discussed in Supplement B. The susceptibility S(0) and correlation length ξ are depicted in Fig. A1b-d for the three temperatures 12, 20.5, and 27.2°C. The solid lines represent S(0), which was evaluated in accordance with (Eq. (5)) from the first derivative of n(T,P) with respect to pressure ($\left.\partial\text{n/}\partial\text{P}\right|_{\text{V,T}}$). The necessary parameters and number density n(T,P) of CO_2_ were taken from Table 1 and ref [[[1]](#endnote-1)], respectively. The coincidence of SANS and n(T,P) is good. In some cases, we had to slightly adapt the SANS experimental temperature with respect to the peak of S(0) from n(T,P) as a reference representing the gas-liquid transition and Widom lines above T_C_. We also had to subtract the Placzek correction factor of 0.25 from the scattering data, which is in the order of S(0) at large pressure fields (Eqs. (2) and (3)).

*A2. Thermal fluctuations – Supercritical regime - Widom line*

Fig. A2 shows susceptibility (S(0)) and correlation length (ξ) for several temperatures in the gas-supercritical regime. Fig. A2a shows scattering data in a Zimm representation determined along the 52 °C isothermal pathway. At 150 bar and below (not shown), the data follow the Ornstein-Zernike (OZ) law (Eq. (1)) over the whole Q range providing the extrapolated scattering cross-section at Q=0, i.e. the susceptibility (dΣ/dΩ(0) and S(0)) as well as the correlation length (ξ) of the thermal density fluctuations. Above 150 bar, we observe a continuously increasingly deviation from OZ-law behavior at low Q representing the formation of new scattering centers of a larger size, indicating the formation of a new phase as will be discussed in the next section.

The susceptibility (S(0)) and correlation length (ξ) derived from the OZ fit (depicted as solid spheres) and S(0) evaluated on the basis of $\left.\partial\text{n/}\partial\text{P}\right|_{\text{V,T}}$are plotted in Fig. A2 b-f. Both values of S(0) show good quantitative agreement as already demonstrated in the previous section. The peaks of S(0) and ξ indicate the Widom line, which is plotted in the CO_2_ phase diagram of Fig.1 showing a

**Table A**. T_Widom_ = A + B×P + C×P^2^ + D×P^3^ polynomial describing the Widom line in SC-CO_2_

| A [°C] | B [10^-2^ °C/bar] | C [10^-4^ °C/bar^2^] | D [10^-7^ °C/bar^3^] |
| --- | --- | --- | --- |
| 1.47±0.025 | 1.7±0.17 | - 1.6±0.29 | 6.22±1.74 |

| a) | b) |
| --- | --- |
| c) | d) |
| e) | f) |
| **Fig. A2.** SANS data on thermal fluctuations in the supercritical regime. a) SANS data measured at 55°C in a Zimm representation beyond the Widom line. b) - f) Susceptibility and correlation length between T = 34.5 and 70 °C. The solid lines represent the susceptibility, S(0), evaluated from the mass density versus pressure as taken from [1]. | |

distinct shift to larger pressures with increasing temperature. The Widom line was fitted with a third-order polynomial in pressure, whose parameters are presented in Table A. This fit is in good agreement with an analysis in [[[2]](#endnote-2)], but at 1.6 and 2.7 K higher temperatures at 110 bar and 120 bar, respectively.

1. **Droplet formation in liquid and supercritical CO_2_**

The formation of droplets was first observed in SC-CO_2_ at 45 °C for hydrostatic pressures above 120 bar [[[3]](#endnote-3)]. Indications of droplet formation for other temperatures also became visible in Figs. A1a and A2a from the deviation of the scattering of the thermal density fluctuations (Eq. (1)). The scattering cross-section of droplets, $\text{ΔdΣ/dΩ(Q)}$, was determined by subtracting the Zimm scattering (dΣ/dΩ_Z_(Q)) from the total scattered intensity according to $\text{dΣ/dΩ(Q)} \text{-} \text{dΣ/dΩ}_{\text{Z}}\text{(Q)}$ as visualized in Fig. 2. We have already mentioned that the scattering probability of the largest ΔdΣ/dΩ(Q) at T = 52 °C in Fig. B2c is calculated between 0.5×10^-5^ and 1.3×10^-5^ (200 and 450 bar), which is extremely low and explains the in part large error bars. Low scattering probability means low droplet volume fraction and / or difference in number density (Δn/n_0_) of the droplet and fluid phase, as we will discuss later.

*B1. Droplet formation beyond the gas-liquid line.*

Fig. A1 shows that the susceptibility and correlation length of the thermal density fluctuations in the gas and liquid regimes becomes greatest along the gas-liquid transition line. The corresponding scattering of droplets, i.e. ΔdΣ/dΩ(Q), is plotted in Fig. B1. At 12 and 20.5 °C, we observe the onset of droplet formation at the gas-liquid line, whereas at 27.2°C (near T_C_= 31 °C) at a slightly higher pressure of ~90 bar this position is identified with the Frenkel line. In all cases, we observe the formation of spherical droplets with an average radius of (43±2) Å (Fig. 3a) and a finite step-wise increase of ΔdΣ/dΩ(0), which is characteristic of first-order phase transitions. The scattering data are depicted in the upper part of Fig. B1a, c, e together with the fitted form factor of spheres (Eq. (7)) shown as solid lines. Increasing pressure transforms ΔdΣ/dΩ(Q) into larger values and finally into a rod-like structure as confirmed by the straight line of Q^-1^ power law behavior in the lower parts of Fig. B1a, c, e ([[[4]](#endnote-4)] (p162)). Furthermore, we identified the shape of

| a) | b) |
| --- | --- |
| c) | d) |
| e) | f) |
| **Fig B1.** **Scattering from droplets ($\text{ΔdΣ/dΩ(Q)}$) in the gas-liquid regime**. Up to about 125 bar droplets are of a spherical shape and above this pressure of a rod-like shape as follows from the fit of the corresponding form factors plotted as solid lines. The corresponding parameters of the fits are depicted in b, d, and f. | |

the rod-like droplets from the scattering curves by fitting with the corresponding form factor of the rods (Eq. (8)) plotted as solid lines

The corresponding fit parameters of the scattering data, i.e. $\text{ΔdΣ/dΩ(0)}$, the radius (R_sp_) of spheres as well as the cross-section (R_rod_) and length (L_rod_) of the rods, are depicted in Fig. B1b, d, f. A reliable $\text{ΔdΣ/dΩ(0)}$ was determined for all individual spherical droplets, but for rod-like droplets only for a sufficiently small length (L_rod_). In these cases, we performed the fit taking a constant length of the rods of 1000 Å, which leads to a correct cross-section due to the factorization of R_rod_ and L_rod_ in the form factor of Eq. (8). At (43±2) Å (Fig. 3a), the radii of the spheres are almost the same for all three temperatures and are slightly larger than the radius of the rod cross-section (32±2) Å (Fig. 3b), i.e. R_sp_/R_rod_ = (1.34±0.11).

*B2. Droplet formation beyond the Frenkel line - Isothermal pathway*

Here, we present the SANS results on formation droplets in the supercritical region occurring above the Frenkel line. Fig. A2 shows the corresponding SANS data of the thermal density fluctuations. The data measured along the isothermal pathway at 34.5 °C in Fig. B2a and b show two stages of droplet formation. In the first step, again spheres of increasing R_sp_. between 43 and 50 Å are found when pressure from 110 to 180 bar is applied. Above 190 bar, rods of a cross-section with an average radius of R_rod_ = (38.2±0.6) Å become visible, which at 450 bar increases to R_rod_ = (53±3) Å. Fig. B2c and d show data measured at 52 °C. The scattering data in Fig. B2c show spherical scattering due to fitting with the spherical form factor (Eq.(6)) displaying (Fig. B2d) a maximal radius of (61.5±1.2) Å accompanied by linearly increasing $\text{ΔdΣ/dΩ(0)}$. Above 325 bar, the droplets transform into a rod-like shape as seen from the data in the lower part of Fig. B2c. The corresponding parameters display a constant cross-section of about (45±2) Å, which is about 25 % smaller than the largest radius of the spheres, but which shows an increasing length (L_rod_) of the rods together with an exponentially increasing $\text{ΔdΣ/dΩ(0)}$ for all pressure fields.

The SANS data in Fig. B3 measured at 56 and 66 °C display different behavior. Spherical droplets are formed beyond the Frenkel line at (173±15) bar and (192±6) bar, respectively, showing a fairly constant radius and linearly increasing ΔdΣ/dΩ(0). Above ~325 bar, a transition to droplets of globular morphology is observed as depicted in the lower part of Fig. B3 a and c. Globular droplets do not follow the form factor of spheres (Eq. (7)) at large Q but have to be fitted with Eq. (9), which combines Guinier’s and Porod’s laws [[[5]](#endnote-5)]. We find globular droplets for pressure fields along 56 °C up to 450 bar but not for 66°C at 450 bar, where we observed a further transition to rod-like droplets, as visualized in the lower part of Fig. B3c. It remains to be mentioned that Fig. B3b shows SANS parameters versus pressure (solid spheres) from 100 to 450 bar as well as the reverse (open spheres) of declining pressure from 450 to 200 bar demonstrating the fast process of

| a) | b) |
| --- | --- |
| c) | d) |
| **Fig B2.** **Scattering data in the supercritical regime**. **a, b)** At 34.5 °C spherical droplets are observed above 105 bar transforming into rod-like droplets at 190 bar in a similar way as observed at the lower temperatures in Fig. B1. **c,d)** At 52 °C and below ~325 °C the droplets transform from a spherical to rod-like shape. In contrast to lower temperatures, the droplets continuously increase from zero at the Frenkel line up to about 60 Å before transforming into a rod-like structure. | |

| a) | b) |
| --- | --- |
| c) | d) |
| **Fig B3.** **Scattering data at 56 and 66 °C**. Again formation of spherical droplets with a radius of ~42 Å beyond the Frenkel line. At about 320 bar, a transition from droplet to globular morphology is observed, which at 66 °C transforms into a rod-like shape at 450 bar. | |

droplet equilibration in SC-CO_2_ fluids. The results presented in Supplement B indicate that individual spherical droplets are directly formed at and above the gas-liquid phase transition and Frenkel lines before they are arranged into rod-like structures at higher pressures. Finally, it should be noted once again that the droplet structure represents an equilibrium state within a time resolution of the experiment as demonstrated in Fig. B3b.

*B3. Experiments on Widom and Frenkel lines in the literature.*

The SANS data in Figs. A1 and A2 are consistent with earlier SAXS and X-ray experiments [[[6]](#endnote-6),[[7]](#endnote-7)]. The authors define the Widom line as a “ridge where the correlation length and the density fluctuations become large” and interpret the Widom line as “the boundary of the phase transition with higher-order”. From our understanding of critical phenomena, we do not follow this interpretation as indicated in Sections A1 and A2. Other experiments identified structural changes in SCFs with the Frenkel line. (i) Inelastic X-ray scattering determined the sound velocity characteristic of liquid-like dynamics in SCF oxygen [[[8]](#endnote-8)]. (ii) Acoustic waves combined with MD simulation and inelastic X-ray scattering in SCF argon showed sharply enhanced positive dispersion when crossing a line the authors erroneously identified as the Widom line [[[9]](#endnote-9)]. (iii) Interatomic distances of SCF argon were determined using X-ray diffraction in a Q range from 1 to 6.5 Å^-1^ [[[10]](#endnote-10),[[11]](#endnote-11)]. The authors report a change of molecular distance when passing the Frenkel line, thereby indicating a change of molecular density on both sides of the Frenkel line. The observation of larger n(T,P) above the Frenkel line corresponds to droplet formation, but this experiment was not performed in the correct Q range for their visualization. (iv) Molecular dynamic (MD) simulations undertaken in [[[12]](#endnote-12),[[13]](#endnote-13),[[14]](#endnote-14)] to determine the Frenkel line of SC-CO_2_ are not convincing as is discussed and demonstrated in Fig. 1 of ref. [3].

*B4. Polymorphic transition in one-component liquids.*

The formation of droplets of different shapes in the liquid and supercritical state of CO_2_ above the first-order gas-liquid transition and Frenkel lines, respectively, is the essential result of this paper (Fig. 1). Droplet formation represents a liquid-liquid transition of phases with the same composition but slightly different number density. Such phase decomposition in disordered one-component liquids and glasses appears to be uncommon and is termed polymorphic phase transition. Molecular density is the driving force representing the order parameter in contrast to the chemical potential in multicomponent mixtures. Polymorphism is reviewed in [[[15]](#endnote-15)] for several liquids and by McMillan et al. in [[[16]](#endnote-16),[[17]](#endnote-17),[[18]](#endnote-18)] also for glasses. An example of polymorphism is reported for phosphorous at about 10 kbar and 1000 °C on basis of *in situ* X-ray diffraction and radiography [[[19]](#endnote-19),[[20]](#endnote-20)]. A reversible molecular structural change transforming phosphorous from a dense molecular fluid to a polymeric liquid might explain this form of polymorphism. We do not know such process of reversible structural change of CO_2_.

Liquid-liquid transitions often occur in [supercooled](https://www.linguee.com/english-german/translation/supercooled.html) liquids below the melting line. These studies are difficult to perform because of competing crystallization. A prominent example of polymorphism in [supercooled](https://www.linguee.com/english-german/translation/supercooled.html) liquids is water since on the basis of molecular dynamic (MD) simulation Stanley’s group presented evidence of two distinct liquid phases inside its supercooled state [[[21]](#endnote-21),[[22]](#endnote-22)]. The starting point for these studies was the maximum of mass density at 4 °C and the minimum of isothermal compressibility at 46 °C. These two anomalies were explained on the basis of the coexistence of two liquid phases of different number density implying a second critical point at T ≈ 200 K and P ≈ 1.5 kbar. This critical point was confirmed quite recently at T ≈ 197 K and P ≈ 1.80 kbar [[[23]](#endnote-23)]. The authors explain the liquid-liquid transition on the basis of an isotropic double-step inter-atomic pair potential as discussed in the second chapter of [[[24]](#endnote-24)]. Palmer et al. (ref. [[[25]](#endnote-25)]) confirmed the “unambiguous evidence” of a liquid–liquid transition of water in response to a study alternatively proposing a liquid–crystal transition [[[26]](#endnote-26)].

Stoiljković’s group followed an independent approach in explaining the phase diagram of SC-ethylene on the basis of an interaction potential, which was formulated in the 18^th^ century by the Jesuit priest R. J. Boscovich [[[27]](#endnote-27)]. Boscovich (1711-1787) was a polymath born in Dubrovnik (Croatia) and educated as a physicist, astronomer, mathematician, philosopher, diplomat, poet, and theologian [[[28]](#endnote-28),[[29]](#endnote-29)]. This interaction potential shows several minima and therefore equilibrium distances of the molecules and was able to identify the Widom line as well as the percolation threshold. This potential corresponds to a certain extent to the proposed interaction potential applied by Stanley’s group [22].

1. **Structural parameters of the droplets**

**Table C1.** Parameters of spherical droplets with relative difference $\text{Δn/n}_{0}$ of the number density of droplet and fluid assuming a constant droplet volume fraction of Φ = 1%. Q2 was determined from the form factor.

| T [°C] | Pressure  [bar] | n_0_(P,T)  [10^22^ cm^-3^] | $\text{ρ}_{\text{CO}_{2}}$ [10^10^ cm^-2^] | R_sp_ [Å] | ΔdΣ/dΩ(0)  [10^-2^ cm^-1^] | Q2  [10^17^ cm^-4^] | $\text{Δn/n}_{0}$ [%]  (Φ = 0.01) |
| --- | --- | --- | --- | --- | --- | --- | --- |
| 12 | 48 | 1.159 | 2.116 | 44.6±3 | 0.39±0.04 | 1.65 | 4.34 |
|  | 65 | 1.194 | 2.180 | 42.3±1.3 | 0.51±0.03 | 2.22 | 4.88 |
|  | 80 | 1.217 | 2.222 | 46.9±0.7 | 0.57±0.02 | 2.08 | 4.64 |
|  | 100 | 1.243 | 2.270 | 42.7±0.6 | 0.73±0.02 | 3.49 | 5.88 |
| 20.5 | 65 | 1.082 | 1.976 | 40.7±1.5 | 0.26±0.02 | 1.42 | 4.32 |
|  | 80 | 1.126 | 2.056 | 42.3±1.1 | 0.31±0.02 | 1.46 | 4.20 |
|  | 100 | 1.166 | 2.129 | 43.1±1.1 | 0.44±0.03 | 1.99 | 4.74 |
| 27.2 | 100 | 1.095 | 1.999 | 42.2±1.2 | 0.36±0.05 | 1.79 | 4.78 |
| 34.5 | 110 | 1.025 | 1.872 | 44.3±4.3 | 0.32±0.06 | 1.38 | 4.48 |
|  | 120 | 1.056 | 1.928 | 39.4±1.7 | 0.34±0.02 | 1.54 | 4.60 |
|  | 130 | 1.081 | 1.974 | 43.4±2.2 | 0.39±0.03 | 1.75 | 4.79 |
|  | 140 | 1.101 | 2.010 | 46±1.2 | 0.48±0.03 | 1.87 | 4.87 |
|  | 150 | 1.120 | 2.044 | 45.4±1.8 | 0.49±0.04 | 1.95 | 4.89 |
|  | 160 | 1.136 | 2.074 | 47.2±1.1 | 0.65±0.03 | 2.31 | 5.24 |
|  | 180 | 1.165 | 2.126 | 49.2±1 | 0.64±0.02 | 1.92 | 4.66 |
| 52 | 200 | 1.06 | 1.93 | 49 ± 3 | 0.35±0.02 | 1.08 | 3.85 |
|  | 250 | 1.13 | 2.06 | 56 ± 3 | 0.70±0.03 | 1.48 | 4.23 |
|  | 300 | 1.18 | 2.16 | 62 ± 2 | 1.28±0.03 | 2.12 | 4.82 |
| 56 | 200 | 1.024 | 1.87 | 43.2±1.2 | 0.23±0.01 | 1.05 | 3.92 |
|  | 250 | 1.102 | 2.013 | 40.6±0.4 | 0.55±0.01 | 3.02 | 6.18 |
|  | 300 | 1.158 | 2.114 | 43.2±0.6 | 0.83±0.03 | 3.98 | 6.75 |
| 66 | 200 | 0.937 | 1.711 | 23±12 | 0.06±0.02 | 0.31 | 2.31 |
|  | 250 | 1.036 | 1.892 | 41.8±0.7 | 0.34±0.01 | 1.68 | 4.90 |
|  | 300 | 1.101 | 2.010 | 41.4±0.5 | 0.68±0.02 | 3.42 | 6.58 |

**Table C2.** Parameters of droplets formed of rod-like shape. The relative difference of $\text{Δn/n}_{0}$ is determined from according to Eqs. (10) and (11) from Q2.

| T [°C] | Pressure  [bar] | n_0_(P,T)  [10^22^ cm^-3^] | $\text{ρ}_{\text{CO}_{2}}$ [10^10^ cm^-2^] | R_rod_ [Å] | L_rod_ [Å] | ΔdΣ/dΩ(0)  [10^-2^cm^-1^] | Q2  [10^17^cm^-4^] | $\text{Δn/n}_{0}$[%]  (Φ = 0.01) |
| --- | --- | --- | --- | --- | --- | --- | --- | --- |
| 12 | 150 | 1.292 | 2.359 | 27.1± 0.9 | Assumed  1000 | 6.66±0.12 | 3.89 | 5.98 |
|  | 200 | 1.330 | 2.429 | 29.3± 1.0 |  | 6.58±0.13 | 3.85 | 5.83 |
|  | 250 | 1.360 | 2.483 | 30.4± 1.2 |  | 5.82±0.13 | 3.16 | 5.12 |
|  | 300 | 1.386 | 2.531 | 29.9± 1.5 |  | 4.58±0.13 | 2.87 | 4.82 |
|  | 350 | 1.409 | 2.573 | 29.1± 1.9 |  | 3.44±0.12 | 2.32 | 4.14 |
|  | 400 | 1.430 | 2.611 | 31.6± 2.3 |  | 3.05±0.13 | 1.80 | 3.68 |
|  | 450 | 1.448 | 2.644 | 31.7± 2.9 |  | 2.37±0.13 | 1.76 | 3.59 |
| 20.5 | 150 | 1.233 | 2.251 | 26.7±0.7 | Assumed  1000 | 7.19±0.17 | 3.52 | 5.96 |
|  | 200 | 1.278 | 2.334 | 28.6±0.8 |  | 7.60±0.30 | 4.06 | 6.25 |
|  | 250 | 1.314 | 2.399 | 32.8±1.0 |  | 6.79±0.21 | 3.65 | 5.75 |
|  | 300 | 1.344 | 2.454 | 32.5±1.0 |  | 6.69±0.21 | 3.34 | 5.34 |
|  | 350 | 1.370 | 2.502 | 33.0±1.3 |  | 5.14±0.20 | 2.82 | 4.84 |
|  | 400 | 1.393 | 2.544 | 32.9±1.5 |  | 4.17±0.19 | 2.36 | 4.36 |
|  | 450 | 1.414 | 2.582 | 33.4±1.9 |  | 3.17±0.18 | 1.84 | 3.76 |
| 27.2 | 150 | 1.182 | 2.158 | 34.3±0.4 | 64±4 | 0.76±0.03 | 3.26 | 5.98 |
|  | 200 | 1.237 | 2.259 |  | 107±7 | 1.14±0.06 | 3.97 | 6.31 |
|  | 250 | 1.278 | 2.334 |  | 145±12 | 1.36±0.09 | 4.26 | 6.33 |
|  | 300 | 1.811 | 3.307 |  | 203±30 | 1.43±0.20 | 3.24 | 5.38 |
|  | 350 | 1.339 | 2.445 |  | 184±26 | 1.26±0.16 | 3.15 | 5.19 |
|  | 400 | 1.364 | 2.491 |  | 228±60 | 1.13±0.27 | 2.42 | 4.47 |
|  | 450 | 1.386 | 2.531 |  | 158±25 | 0.68±0.09 | 2.02 | 4.02 |
| 34.5 | 300 | 1.274 | 2.326 | 38.2±0.6 | 1000 | 7.40±0.12 | 2.83 | 5.18 |
|  | 350 | 1.305 | 2.383 |  |  | 7.69±0.13 | 2.94 | 5.15 |
|  | 400 | 1.332 | 2.432 |  |  | 6.00±0.49 | 2.28 | 4.44 |
|  | 450 | 1.356 | 2.476 | 52.8±2.8 | 1200±541 | 15.6±7.2 | 5.64 | 6.86 |
| 52 | 350 | 1.220 | 2.228 | 45.7±1.3 | 189±16 | 1.83±0.12 | 2.67 | 5.25 |
|  | 400 | 1.254 | 2.290 | 41±1.5 | 523±34 | 4.49±0.22 | 2.92 | 5.34 |
|  | 450 | 1.283 | 2.343 | 48±1.4 | ------- | ------- | 2.18 | 4.51 |
| 66 | 450 | 1.224 | 2.235 | 32.6±0.6 | 416±119 | 3.4±0.9 | 4.10 | 6.48 |

**Table C3.** Parameters of droplets of globular morphology

| T [°C] | Pressure  [bar] | n_0_(P,T)  [10^22^ cm^-3^] | $\text{ρ}_{\text{CO}_{2}}$ [10^10^ cm^-2^] | R_g_ [Å] | ΔdΣ/dΩ(0)  [10^-2^cm^-1^] | Q2  [10^17^cm^-4^] | Δn/n_0_ [%]  (Φ = 0.01) |
| --- | --- | --- | --- | --- | --- | --- | --- |
| 56 | 350 | 1.200 | 2.191 | 42.1±1.4 | 1.22±0.09 | 6.91 | 8.54 |
|  | 400 | 1.236 | 2.257 | 42.9±1.3 | 1.27±0.09 | 7.00 | 8.40 |
|  | 450 | 1.266 | 2.312 | 48.7±1.2 | 1.38±0.05 | 6.03 | 7.56 |
| 66 | 350 | 1.150 | 2.100 | 38.5±0.53 | 1.03±0.03 | 7.22 | 9.11 |
|  | 400 | 1.190 | 2.173 | 41.5±0.13 | 1.3±0.03 | 7.71 | 9.10 |

**References**

1. [] Lemmon, E.W., McLinden, M.O. & Friend, D.G. "Thermophysical Properties of Fluid Systems" in **NIST Chemistry WebBook, NIST Standard Reference Database Number 69**, Eds. Linstrom, P.J & Mallard, W.G. National Institute of Standards and Technology, Gaithersburg MD, 20899, <https://doi.org/10.18434/T4D303>, (retrieved October 15, 2019). [↑](#endnote-ref-1)
2. [] Imre, A., Ramboz, C., Kraska, T. & Deiters, U.K. Anomalous fluid properties of carbon dioxide in the supercritical region – Application to geological CO_2_ storage and related hazards, *Environmental Earth Sciences*, **73**, 4373-4384 (2015). [↑](#endnote-ref-2)
3. [] Pipich, V. & Schwahn, D. Densification of Supercritical Carbon Dioxide (CO_2_) accompanied by Droplet Formation when Passing the Widom Line. Phys. Rev. Lett. **120**, 145701 (2018). [↑](#endnote-ref-3)
4. [] Roe, R.J. *Methods of X-Ray and Neutron Scattering in Polymer Science* (University Press, Oxford, 2000). [↑](#endnote-ref-4)
5. [] Beaucage, G. Approximations leading to a unified exponential power-law approach to small-angle scattering, *J. Appl. Cryst.* **28**, 717-728 (1995). [↑](#endnote-ref-5)
6. [] Nishikawa, K., Tanaka, I. & Amemiya, Y. Small-Angle X-ray Scattering of Supercritical Carbon Dioxide, *J. Chem*. **100**, 418-421 (1996). [↑](#endnote-ref-6)
7. [] Morita, T., Nishikawa, K., Takematsu, M., Iida, H. & Furutaka, S. Structure Study of Supercritical CO_2_ near Higher-Order Phase Transition Line by X-ray Diffraction, *J. Chem*. **101**, 7158-7162 (1997). [↑](#endnote-ref-7)
8. [] Gorelli, F., Santoro, M., Scopigni, T., Krisch, M. & Ruacco, G. Liquidlike behavior of supercritical fluids, Phys. Rev. Lett. **97**, 245702 (2006). [↑](#endnote-ref-8)
9. [] McMillan, P.F. & Stanley, H.E. Going supercritical, Nature Physics **6**, 479-480 (2010). [↑](#endnote-ref-9)
10. [] Bolmatov, D., Brazhkin, V.V., Fomin, Yu.D., Ryzhov, V.N. & Trachenko, K. Evidence for structural crossover in the supercritical state, *J. Chem. Phys*. **139**, 234501 (2013). [↑](#endnote-ref-10)
11. [] Bolmatov, D. *et al*. The Frenkel line: A direct experimental evidence for the new thermodynamic boundary, *Scientific Reports* **5**, Article number: 15850 (2015). [↑](#endnote-ref-11)
12. [] Brazhkin, V.V. *et al.* The Frenkel line and supercritical technologies, *Russian Journal of Physical Chemistry* *B* **8**, 1087-1094 (2014). [↑](#endnote-ref-12)
13. [] Fomin, Yu.D., Ryzhov, Y.N., Tsiok, E.N. & Brazhkin, V.V. Thermodynamic properties of supercritical carbon dioxide: Widom and Frenkel lines, *Phys. Rev. E* **91**, 022111 (2015). [↑](#endnote-ref-13)
14. [] Yang, C., Brazhkin, V.V., Dove, M.T. & Trachenko, K. Frenkel line and solubility maximum in supercritical fluids, *Phys. Rev. E* **91**, 012112 (2015). [↑](#endnote-ref-14)
15. [] Liquid Polymorphism, *Advances in Chemical Physics*, Volume 152, Stanley, H. E. (Editor), (John Wiley & Sons, New Jersey 2013). [↑](#endnote-ref-15)
16. [] Poole, P.H., Grande, T., Angell, C.A. & McMillan, P.F. Polymorphic phase transitions in liquids and glasses, *Science* **275**, 322-323 (1997). [↑](#endnote-ref-16)
17. [] McMillan, P.F. Polyamorphic transformations in liquids and glasses, *J. Mater. Chem*. **14**, 1506 – 1512 (2004). [↑](#endnote-ref-17)
18. [] McMillan, P.F. *et al.* Polyamorphism and liquid–liquid phase transitions: challenges for experiment and theory, *J. Phys.: Condens. Matter* **19**, 415101 (2007). [↑](#endnote-ref-18)
19. [] Katayama, Y. *et al.* A first-order liquid-liquid phase transition in phosphorus, *Nature* **403**, 170-173 (2000). [↑](#endnote-ref-19)
20. [] Katayama, Y. *et al.* Macroscopic separation of dense fluid phase and liquid phase of phosphorus, *Science* **306**, 848-851 (2004). [↑](#endnote-ref-20)
21. [] Poole, P.H., Sciortino, F., Essmann, U. & Stanley, H.E. Phase behavior of metastable water, *Nature* **360**, 324-328 (1992). [↑](#endnote-ref-21)
22. [] Buldyrev, S.V*. et al*. Models for a liquid–liquid phase transition, *Physica A* **304**, 23-42 (2002). [↑](#endnote-ref-22)
23. [] Mallamace, F., Corsaro, C., Mallamace, D., Fazio, E. & Chen, S.H. Some considerations on the water polymorphism and the liquid-liquid transition by the density behavior in the liquid phase, *J. Chem. Phys*. 151, 044504 (2019). [↑](#endnote-ref-23)
24. [] Ben-Naim, A. *Molecular theory of water and aqueous solutions*, Part I: Understanding water, (World Scientific Publishing. Singapore 2009). [↑](#endnote-ref-24)
25. [] Palmer, J.C. *et al*. Metastable liquid–liquid transition in a molecular model of water, *Nature* **510**, 385-388 (2014). [↑](#endnote-ref-25)
26. [] Limmer, D.T. & Chandler, D. The putative liquid-liquid transition is a liquid-solid transition in atomistic models of water, *J. Chem. Phys*. **135**, 214504 (2011). [↑](#endnote-ref-26)
27. [] Stoiljković, D. & Jovanović, S. Compression, supramolecular organization and free radical polymerization of ethylene gas**,** *J. Polyolefins* **6**, 23-41 (2019). [↑](#endnote-ref-27)
28. [] Boscovich, R.J. *Theoria philosophiae naturalis*. Venetiis, Ex Typographia Remondiniana (1763); *A Theory of Natural Philosophy* (Open Court Publishing Company, Chicago - London 1922) (<http://archive.org/details/theoryofnaturalp00boscrich>). [↑](#endnote-ref-28)
29. [] Ullmaier, H. *Puncta, particulae et phaenomena. Der dalmatische Gelehrte Roger Boscovich und seine Naturphilosophie* (Wehrhahn Verlag, Hannover-Laatzen 2005). [↑](#endnote-ref-29)
